# Binge Alcohol Exposure Causes Neurobehavioral Deficits and GSK3β Activation in the Hippocampus of Adolescent Rats

**DOI:** 10.1038/s41598-018-21341-w

**Published:** 2018-02-15

**Authors:** Zhe Ji, Lin Yuan, Xiong Lu, Hanqing Ding, Jia Luo, Zun-Ji Ke

**Affiliations:** 1 Department of Biochemistry, Shanghai University of Traditional Chinese Medicine, 1200 Cailun Road, Shanghai, 201203 China; 2 Translational Medicine Research Center, Ruijin Hospital North, Shanghai Jiao Tong University School of Medicine, Shanghai, 201821 China; 3 Department of Pharmacology and Nutritional Sciences, University of Kentucky College of Medicine, Lexington, Kentucky 40536 USA

## Abstract

Heavy alcohol exposure causes profound damage to the adolescent brain, particularly the hippocampus, which underlie some behavioral deficits. However, the underlying molecular mechanisms remain inconclusive. The current study sought to determine whether binge alcohol exposure affects the hippocampus-related behaviors and key signaling proteins that may mediate alcohol neurotoxicity in adolescent rats. Alcohol exposure reduced the number of both NeuN-positive and doublecortin-positive cells in the hippocampus. Alcohol also induced neurodegeneration which was confirmed by ultrastructural analysis by electronic microscopy and was accompanied with the activation of microglia. Binge alcohol exposure impaired spatial learning and memory which was evaluated by the Morris water maze. However, alcohol did not alter the spontaneous locomotor activity which was determined by the open field test. GSK3β is a multi-function serine/threonine protein kinase regulating both neuronal survival and neurogenesis and plays an important role in various neurodegenerative disorders. We have previously shown that GSK3β is a key mediator of alcohol-induced neuron apoptosis in the developing brain. We showed here binge alcohol exposure caused GSK3β activation by inducing dephosphorylation at Ser9 without affecting the phosphorylation of Tyr216 in the hippocampus. Thus, GSK3β may be involved in binge alcohol exposure-induced neuronal damage to the adolescent hippocampus.

## Introduction

Alcohol use in the adolescents has significantly increased in the U.S. as well as other countries during the last 10 years^1–6^. Due to the ongoing process of maturation, the adolescent brain is vulnerable to alcohol abuse and other substance abuse^7^. A potential long-lasting consequence of alcohol use during adolescence is the increased risk of developing alcohol abuse and dependence in adulthood^8,9^. People who begin drinking before the age of 15 are four times more likely to develop alcohol dependence at some time in their lives compared with those who start drinking at the age of 20 or later^10^.

During adolescence, the brain is undergoing maturation. This process involves changes in neurotransmission and plasticity which is accompanied with structural modifications in some brain regions, such as the hippocampus, prefrontal cortex and the limbic system structures^11^. Both clinical and experimental studies have provided evidence showing that alcohol exposure profoundly affected adolescent brain, such as memory impairments^12^, structural alterations^13^ and inflammatory brain damage^14^. However, the underlying mechanisms are not clear. The hippocampus was selected for our study because of its critical role in the learning and memory. Beside the subventricular zone (SVZ) in the forbrain, the hippocampus is another regions in which neurogenesis persist throughout life. In the hippocampus, neural progenitor cells (NPCs) located along the subgranular zone (SGZ) produce new granule neurons in the hippocampal dentate gyrus (DG), which play an important role in spatial learning and memory^15^, In this study, we sought to investigate alcohol-induced neurodegeneration in the hippocampus and associated behavioral deficits using a binge alcohol exposure model.

Glycogen synthase kinase 3 (GSK3), a serine/threonine kinase originally identified as a regulator of glycogen metabolism, is a central component of the Wnt signaling pathway important for proper axis formation during embryonic development^16,17^. There are two highly homologous forms of GSK3 in mammals, GSK3α and GSK3β. Both isoforms are ubiquitously expressed. The expression of GSK3β in the central nervous system is developmentally regulated and localized primarily in neurons^17,18^. GSK3β is unusual in that it is largely regulated by inhibition. One mechanism through which GSK3β can be inactivated is the phosphorylation of serine 9 (Ser9)^16,19^. Numerous protein kinases, such as Akt/protein kinase B (PKB), protein kinase C (PKC), p70 S6 kinase, p90Rsk, and protein kinase A (PKA), can phosphorylate GSK3β at Ser9 and therefore inactivate GSK3β^19^. GSK3β is also activated by phosphorylation at tyrosine 216 (Tyr216). More than 40 proteins are substrates of GSK3β, and these proteins have roles in a wide spectrum of cellular processes, including glycogen metabolism, transcription, translation, cytoskeletal regulation, cell differentiation, proliferation, transformation, and apoptosis^19,20^. GSK3β plays an important role in the regulation of neuronal survival, neuroinflammation, and microglial activation in the CNS^21,22^. We have previously demonstrated that alcohol activates GSK3β which mediates alcohol-induced neurodegeneration in the developing brain^17,23,24^. In this study, we sought to determine whether binge alcohol exposure activates GSK3β in the adolescent brain.

## Results

### Binge alcohol exposure induces neurodegeneration and microglial activation in the hippocampus of adolescent rats

**Figure 1:**
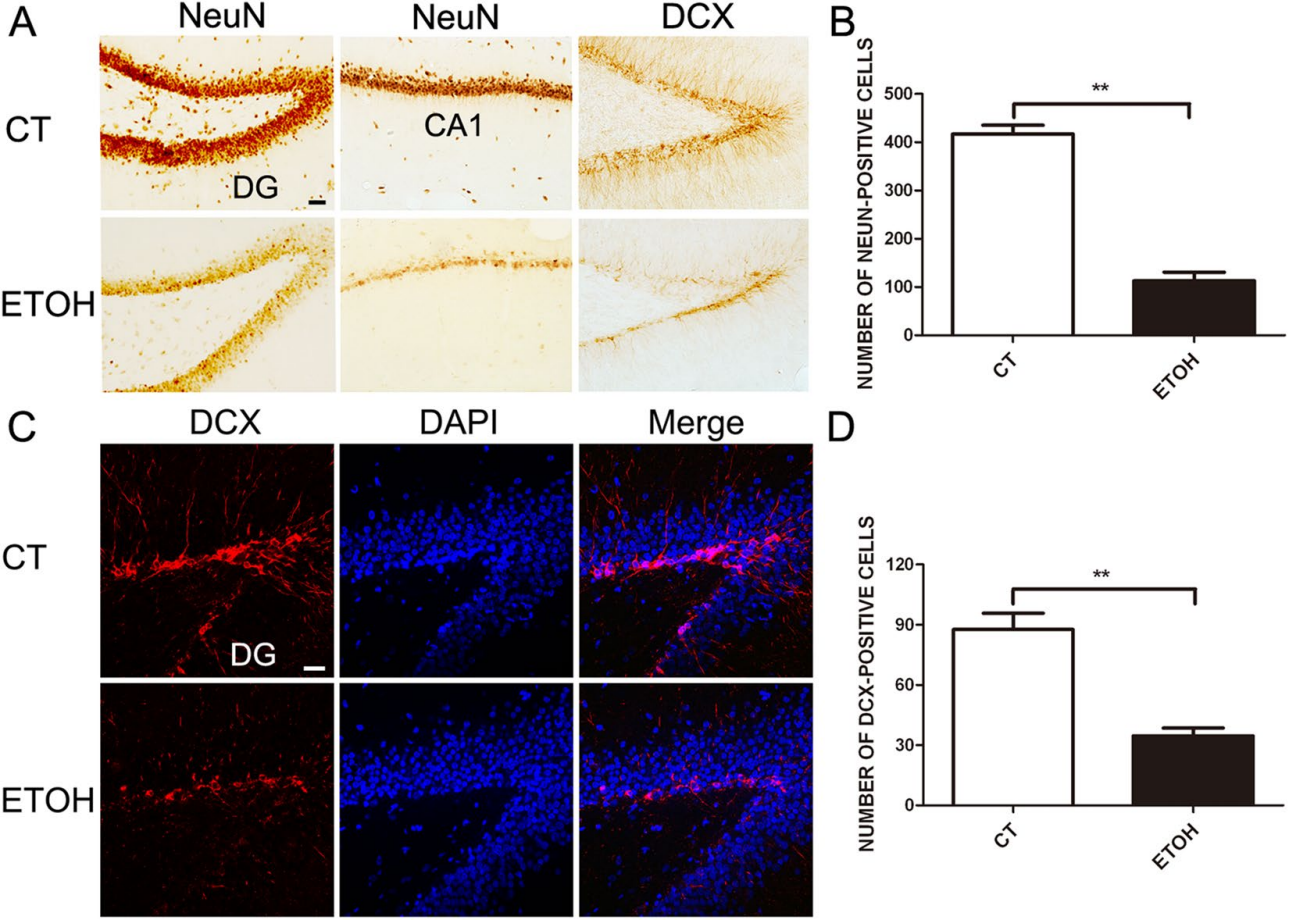
Binge alcohol exposure depletes neurons in the hippocampus of adolescent rats. Adolescent rats were exposed to alcohol every 8 hours for 4 days as described under the Materials and Methods. The neuron and newborn neurons in the DG or CA1 were measure by immunohistochemical (IHC) staining with an antibody directed against NeuN or DCX (**A**). Bars = 50 μm. (**B**) The number of NeuN-positive cells in the hippocampus was quantified using ImageJ software in a DG area. Data were presented as mean ± SEM; CT: 417.0 ± 18.25, ETOH: 112.7 ± 17.9 **p = 0.0003, compared with rats treated with alcohol; n = 5 for each group. Each section was chosen from every 6^th^ section. DCX-positive cells in the dentate gyrus of hippocampus (**C**) were quantified using ImageJ software (**D**). Data were presented as mean ± SEM; CT: 87.67 ± 8.11, ETOH: 34.67 ± 3.84 **p = 0.0041, compared with rats treated with alcohol; n = 5 for each group. Bars = 20 μm.

We first examined the effect of alcohol on neuronal death/proliferation and microglial activation in that hippocampus to validate that this is a relevant paradigm. There were two groups: control (CT) and alcohol-exposed rats (ETOH). There were 35 rats in each group that received gavage for 4 days. In ETOH group, 27 rats showed ataxia and loss of righting reflex symptoms. The body weight was recorded each day; there was no significantly different in body weight between CT and ETOH group after the treatment. The 6 points behavioral intoxication scales were used to determine subsequent doses after the priming dose of 5 g/kg. The average final dose of alcohol was 4.25 g/kg (SD = 0.54). Then we determined whether binge alcohol exposure altered the number of neurons in the adolescent hippocampus. As shown in Fig. 1, alcohol exposure significantly reduced the number of NeuN-positive neurons in the CA1 area of hippocampus (Fig. 1A and B) as well as DCX-positive cells in the DG (Fig. 1C and D). These DCX-positive cells are presumably newly formed neurons.

**Figure 2:**
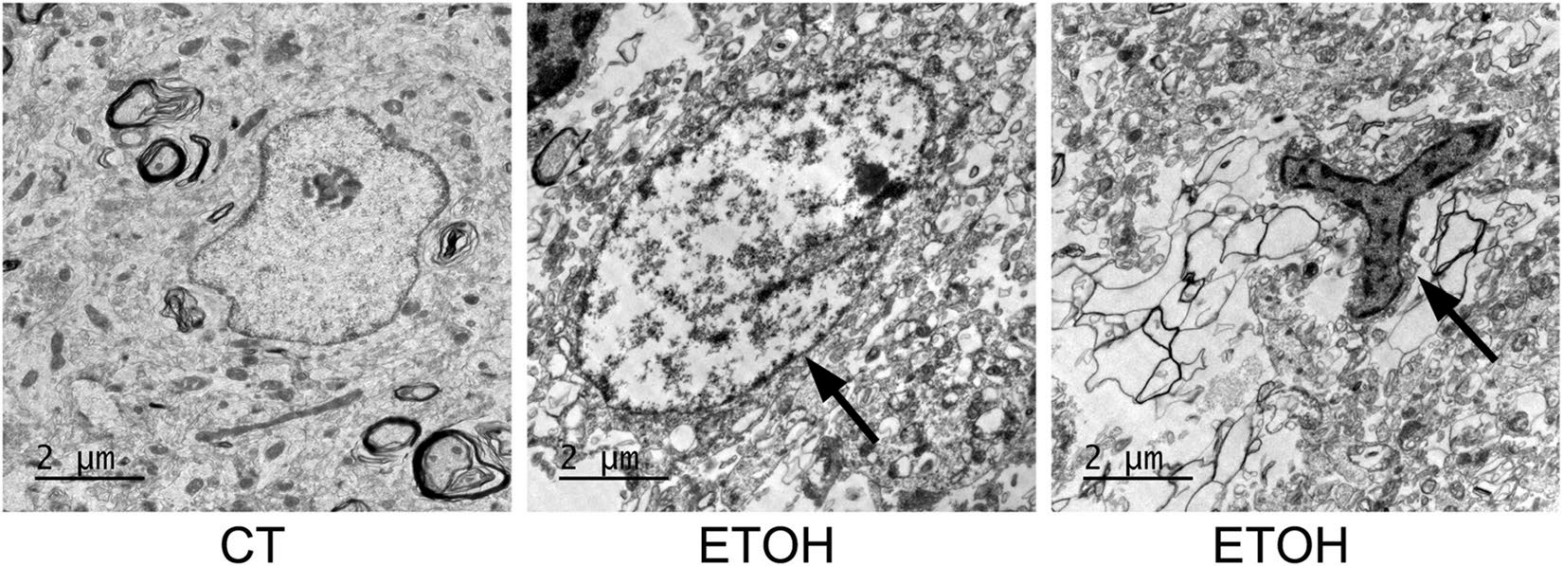
Ultrastructural analysis of alcohol-induced damage in the hippocampus of adolescent rats. Adolescent rats were treated with alcohol and processed as described in Fig. 1. The ultrastructure of the hippocampus was analyzed by electron microscopy (EM). A representative EM image shows damaged neurons (arrow in Fig. 3 middle) and activated microgila in the granular layer of the dentate gyrus (arrow in Fig. 3 right). Bars = 2 μm.

We used electron microscopy (EM) to further evaluate alcohol-induced ultrastructural alterations in the hippocampus. Degenerating neurons were observed in the granular layer (GL) of the DG (Fig. 2). The damaged neurons showed condensation and fragmentation of nuclear chromatin (arrow in Fig. 2 middle panel), as well as numerous vacuoles within the cytoplasm. These vacuoles are comprised of swollen organelles such as mitochondria, Golgi apparatus, and endoplasmic reticulum. There was also partial disruption of the cytoplasm membrane. This is the first report to demonstrate alcohol-induced neurodegeneration in the adolescent hippocampus using EM. In addition, microglia were found adjacent to the damaged neurons (arrow in Fig. 2, right panel).

### Binge alcohol exposure activates microglia in the hippocampus of adolescent rats

**Figure 3:**
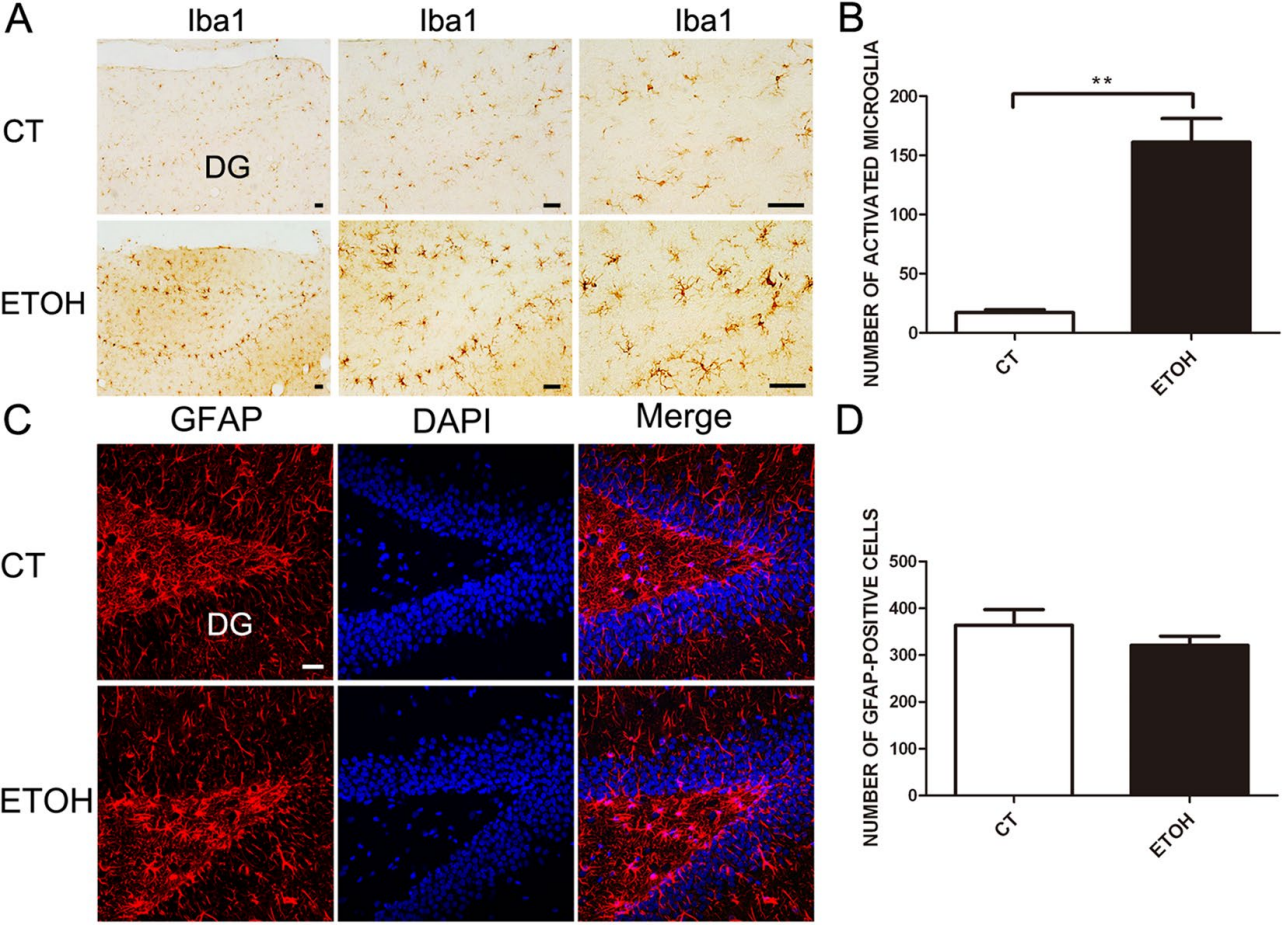
Binge alcohol exposure causes neuroinflammation in the hippocampus of adolescent rats. Adolescent rats were treated with alcohol and processed as describe in Fig. 1. The activation of microglia and astrocytes was examined by IHC and immunofluorescence staining using antibodies directed against Iba-1 (**A**) and GFAP (**C**), respectively. Bars = 50 μm. The number of activated micoglia and astrocytes was determined and shown in panel **B** and **D**, respectively. n = 5 for each group. Data were presented as mean ± SEM; CT: 17 ± 2.65, ETOH: 161 ± 20 **p = 0.0021; CT: 364 ± 33.45, ETOH: 321 ± 19.5 p = 0.3290.

We examined the effect of binge alcohol exposure on microglia and astrocytes. In alcohol-exposed rats, more microglia with an active morphology (larger cell body and thicker processes) was observed compared to the control rats (Fig. 3A and B). However, alcohol had little effect on astrocytes (Fig. 3C and D). These results suggested that binge alcohol exposure activated microglia.

### Binge alcohol exposure impairs spatial memory in adolescent rats

**Figure 4:**
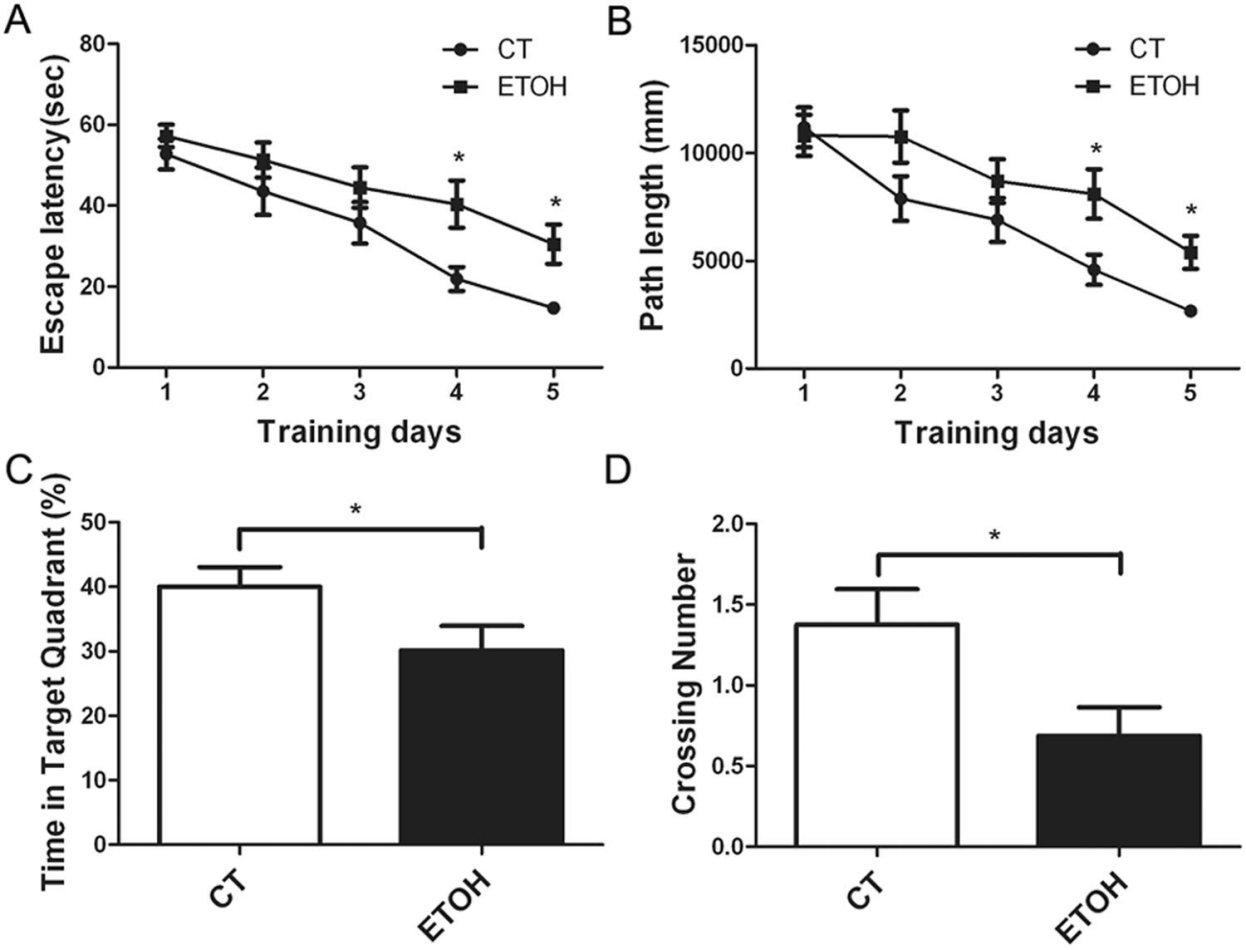
Effect of binge alcohol exposure on the spatial memory of adolescent rats. Adolescent rats received binge alcohol exposure for 4 days as described under the Materials and Methods. Three days after the last alcohol exposure, the rats were trained in the Morris water maze (MWM) and the learning ability and spatial memory were assessed. (**A**) The latency for finding the platform for each trial during the 5 day testing period was measured. (**B**) The swimming distance for finding the platform for each trial during the 5 day testing period was measured. All values are expressed as mean ± S.E.M. n = 10 for each group. *Indicates significant difference from the control group (p < 0.05). (**C**) The ratio of time spent in the target quadrant was determined. Data were presented as mean ± SEM; CT: 40.03 ± 3.02, ETOH: 30.13 ± 3.82 *p = 0.0398; (**D**) The time of crossing the platform was measured. Data were presented as mean ± SEM; CT: 1.375 ± 0.22, ETOH: 0.6875 ± 0.17 *p = 0.0212.

**Figure 5:**
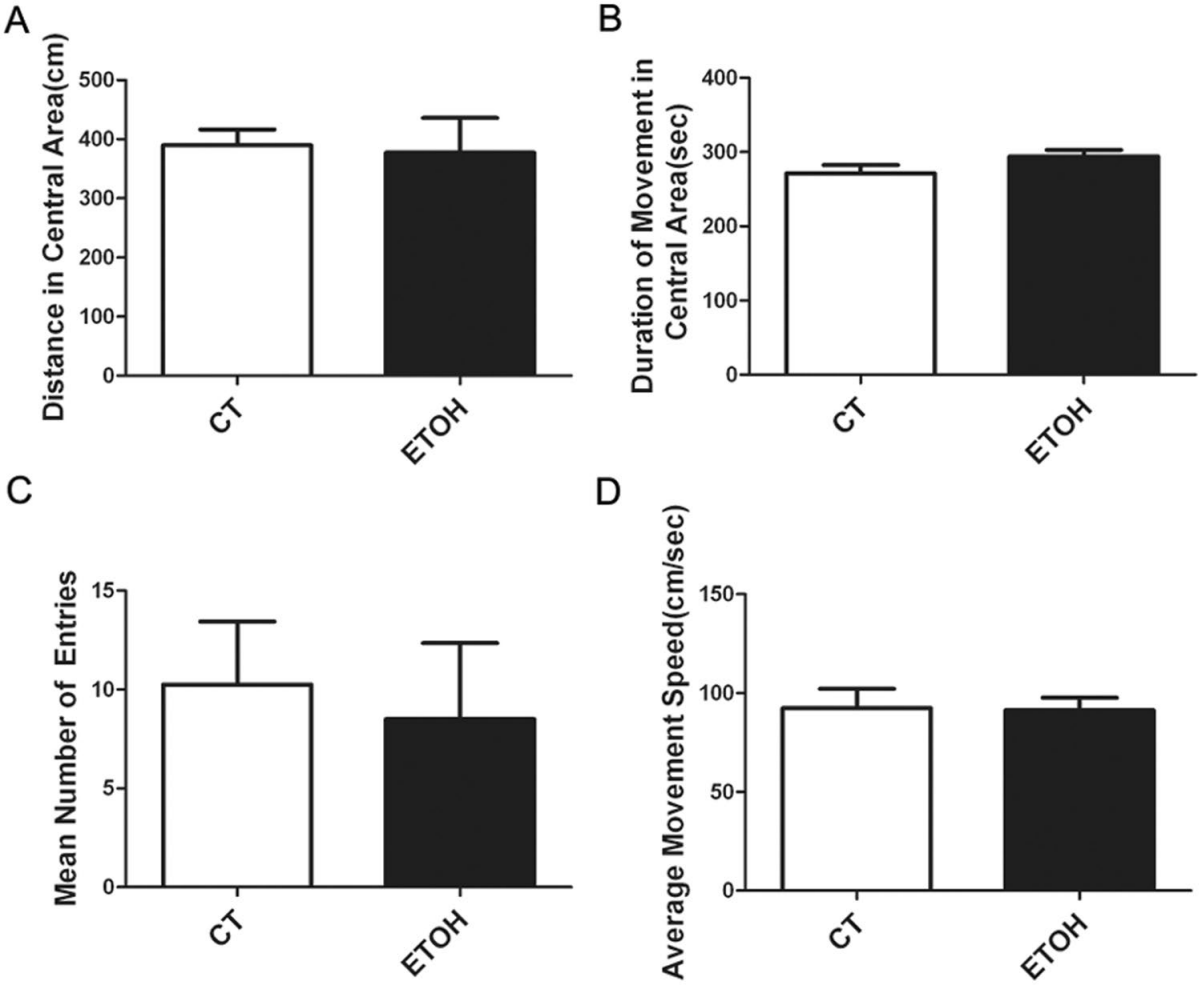
Effect of binge alcohol exposure on the anxiety-related behavior of adolescent rats. Adolescent rats received binge alcohol exposure for 4 days as described under the Materials and Methods. One day after the last alcohol exposure, open field test was performed on these rats to assess the spontaneous locomotor activity. (**A**) The amount of distance in which the rats traveled was measured. (**B**) The relative duration in which rats stayed in the center of the open field was determined. (**C**) The number which the rats entered the center of the open field was calculated. (**D**) The average speed of movement of the rats in the open field test was determined. All values are expressed as mean ± S.E.M, n = 10 for each group.

We used the Morris water maze to assess the effect of binge alcohol exposure on spatial memory. As shown in Fig. 4A and B, all animals showed a progressive decline in the escape latency and distance of swimming during the 5 day-training period. However, alcohol exposure significantly increased the escape latency and distance of swimming on the fourth and fifth training days. For the probe trial, the platform was removed, and the animals were placed in quadrant 2 which was opposite to the target quadrant (quadrant 4). The rats were given 60 s to search the target quadrant. We measured the percentage of time spent in the target quadrant and the number of these rats crossing the area in the original platform location. As shown in Fig. 4C and D, binge alcohol exposure significantly decreased the percentage of time spent in the target quadrant and the number of these rats crossing the area of original platform placement. However, in the open field test, the distance traveled and the time spent in the center of the open field were comparable between two groups. Meanwhile, there was no significant difference in the number of entering the center and the average speed. It appeared that binge alcohol exposure did not alter the anxiety-related behavior of adolescent rats (Fig. 5).

### Binge alcohol exposure activates GSK3β in the adolescent hippocampus

**Figure 6:**
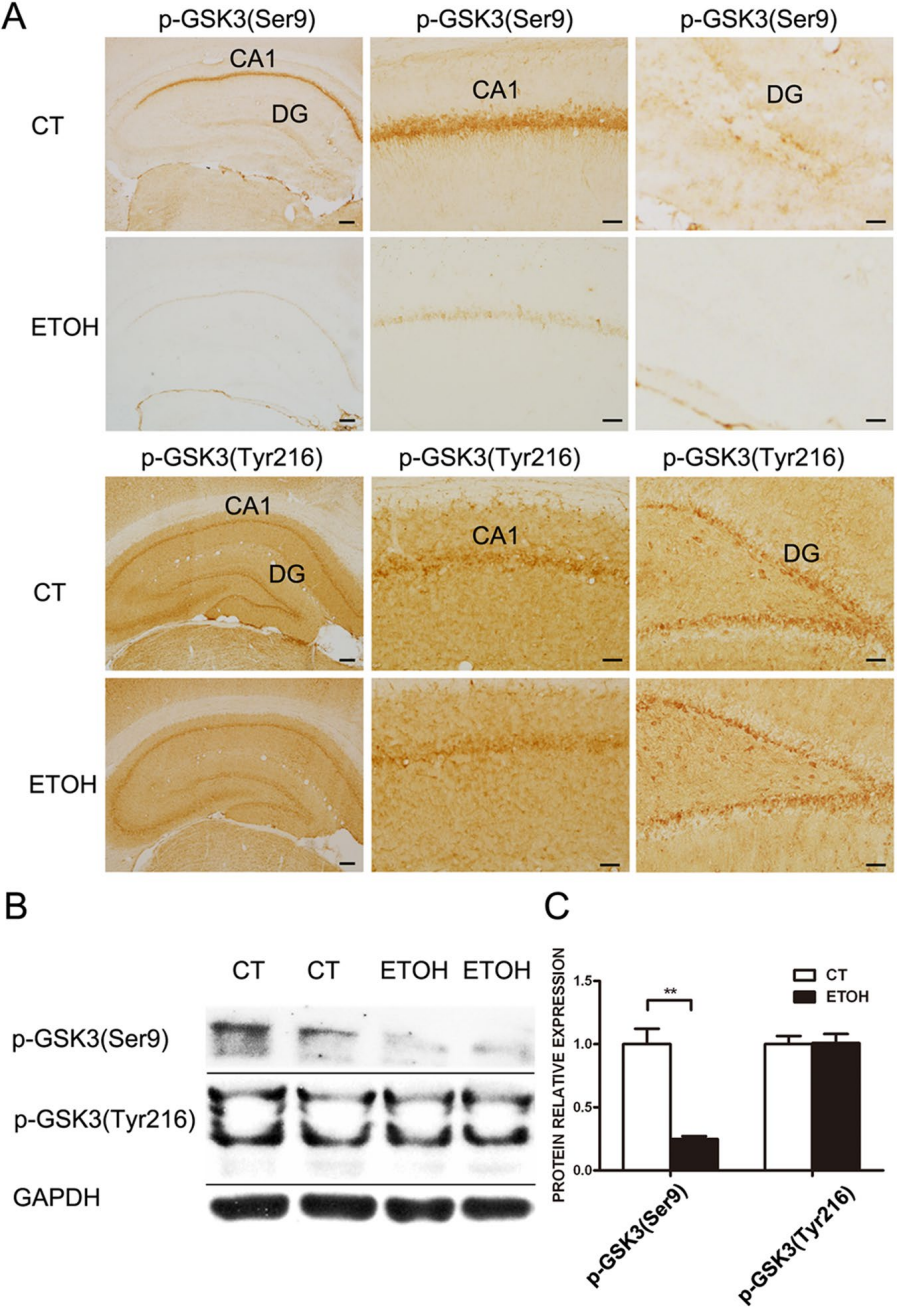
Effect of binge alcohol exposure on GSK3β activation in the hippocampus of adolescent rats. Adolescent rats received binge alcohol exposure for 4 days as described under the Materials and Methods. (**A**) Rats were euthanized by intracardiac perfusion with ice-cold PBS, followed by 4% paraformaldehyde solution. Brain tissues were dissected and subjected to IHC analysis of GSK3β activation. The phosphorylation of GSK3β at Ser9 and Tyr216 were examined by IHC staining using antibodies directed against phosphorylated GSK3β (Ser9 or Tyr216). Bars = 50 μm. (**B**) Hippocampal tissues were dissected and analyzed for the expression of phosphorylated GSK3β (Ser9 or Tyr216) by immunoblotting. (**C**) The relative amounts of phosphorylated GSK3β (Ser9 or Tyr216) were quantified by the densitometry and normalized to the expression of GAPDH. The experiment was replicated three times. Data were presented as mean ± SEM; CT: 109.2 ± 7.65, ETOH: 27.09 ± 1.47 **p = 0.0058.

Since GSK3β is a key mediator of alcohol-induced neurodegeneration in the developing brain and is activated by alcohol exposure during the early postnatal period^23,25^, we sought to determine whether alcohol also activates GSK3β and causes neurodegeneration in the adolescent hippocampus. The activity of GSK3β is negatively regulated by its phosphorylation at Ser9 but positively regulated by its phosphorylation at Tyr216^16^. There was a strong expression of phosphorylated GSK3β (Ser9) in the CA1 in the hippocampus of control adolescent rats, suggesting that the activity of GSK3β was constitutively inactivated (Fig. 6A). Binge alcohol exposure caused a drastic dephosphorylation of GSK3β (Ser9), but had little effect on the phosphorylation at Tyr216 (Fig. 6A). This finding was confirmed by immunoblotting results (Fig. 6B and C), which indicated that alcohol activated GSK3β.

## Discussion

In this study, we used a well-established rat model of 4-days binge alcohol exposure to investigate the cellular mechanisms underlying alcohol-induced damage to the adolescent brain. We showed that binge alcohol exposure causes neurodegeneration and microglial activation in the hippocampus. Alcohol also produced hippocampus-associated behavioral deficits. Alcohol activated GSK3β by inducing Ser9 dephopshorylation in the hippocampus.

We confirmed that this paradigm of binge alcohol exposure significantly reduced the number of both mature neurons and newly formed neurons (DCX-positive) (Fig. 1). The ultrastructural study revealed degenerating neurons in the granular layer of the dentate gyrus which are surrounded by active microglia (Fig. 2). This is the first report to directly demonstrate alcohol-induced neurodegeneration in the adolescent hippocampus using EM. We did not observe an increase in the active form of caspase-3 (data not shown), which was consistent with previous findings showing that alcohol-induced cell death under this paradigm may not be mediated by apoptosis^26^.

Hippocampal volume loss has been observed in adolescent humans with alcohol use disorder (AUDs)^27–29^. The hippocampus is one of two brain regions in which neurogenesis persist throughout life. In the hippocampus, NPCs located along the SGZ produce new granule neurons in the hippocampal dentate gyrus^15^. The continuous generation of new neurons regulates the size and structure of the dentate gyrus granule layer^30^. Both binge and chronic alcohol exposure have been shown to inhibit hippocampal neurogenesis in adolescent rats^26,31,32^. The reduction of DCX-positive cells in the SGZ may reflect either the inhibition of NPC proliferation and death of newly born neurons or both. Indeed, previous studies using the same paradigm provided evidence showing that alcohol inhibits proliferation of NPC in the adolescent hippocampus^33,34^. Our data showed alcohol-induced hippocampal damage is accompanied by the activation of microglia (Fig. 3). The degenerating neurons were surrounded by microglia (Fig. 2), suggesting that that microglia are activated in response to alcohol-induced neurodegeneration to clear damaged neurons. However, it is also possible that alcohol directly targets microglia which initiate a pro-inflammatory response, causing neuronal damage.

We showed that binge alcohol administration by this paradigm resulted in poor water maze escape performance but not locomotor activity in open field test in adolescent rats, indicating impaired spatial learning and memory (Fig. 4). This was consistent with the notion that adolescents are resistant to the motor-impairing and sedative effects of alcohol, but more sensitive to its rewarding and reinforcing properties, compared to adults^35,36^. Alcohol-induced poor water maze performance may result from impaired spatial learning and memory as well as other nonspecific performance factors, such as the disruption of sensorimotor functions^37,38^. In our study, alcohol exposure had little effect on spontaneous locomotor activity (Fig. 5), supporting the hypothesis that deficits in maze performance may result from impaired spatial learning and memory but not due to the dysfunction of spontaneous locomotor activity. Using different alcohol exposure paradigms, Acheson *et al*.^39^ and Markwiese *et al*.^40^ suggested that adolescent alcohol exposure may impair the acquisition of spatial memory in the water maze but not non-spatial memory.

It is unclear whether the effect of binge alcohol exposure during adolescence on spatial learning and memory persists to adulthood. It appears that the effect of alcohol on the behavior of adolescent animals depends on the paradigm of alcohol exposure. For example, Osborne and Butler^41^ investigated the lasting effect of alcohol exposure to peri-adolescent rats. They examined the effects of alcohol on passive avoidance beginning 20 days after treatment, when rats became adults. Alcohol exposure during the peri-adolescence caused impaired avoidance tasks in adult animals. Slawecki and Betancourt^42^ treated adolescent rats with alcohol for 10 consecutive days from postnatal (PD) 30 to PD 40 and then measured alcohol self-administration in adults. They did not find a significant alteration in alcohol self-administration between alcohol-exposed and control rats. White *et al*.^43^ investigated the effect of binge-pattern alcohol exposure on 30-day old rats. In their model, rats were administered alcohol (5.0 g/kg) every 48 hours over a 20-day period. They evaluated the performance of adult rats in an eight-arm radial maze and revealed no difference between alcohol-exposed and control groups.

Glycogen synthase kinase 3β (GSK3β) is a multifunctional serine/threonine kinase. Since GSK3β has diverse substrates ranging from metabolic/signaling proteins and structural proteins to transcription factors, it regulates diverse neuronal functions such as neuronal survival, neurogenesis, differentiation, and plasticity^17,23^. GSK3β is particularly abundant in the developing CNS and is involved in many developmental events in the immature brain, such as neurogenesis, neuronal migration, differentiation and survival^17^. GSK3β also plays an important role in various neurodegenerative disorders^44,45^. In fact, GSK3β may mediate neurodegeneration induced by diverse environmental insults and neurotoxins^23^. The activity of GSK3β is affected by various environmental/cellular insults, such as derivation of nutrients/trophic factors, neuroinflammation, oxidative stress and endoplasmic reticulum stress. We showed here that alcohol can activate GSK3β by inducing the dephosphorylation of GSK3β at serine 9 in the hippocampus (Fig. 6), suggesting that GSK3β may also be involved in alcohol-induced damage to the adolescent hippocampus. We have previously demonstrated that alcohol can activate GSK3β in the developing brain and GSK3β activation mediates alcohol-induced neurodegeneration and disruption of neuronal differentiation^25,46^. Therefore, it appears that some behavioral deficits caused by adolescent exposure to alcohol may be persistent and exhibited in the adulthood, but some may diminish. To further establish the role of GSK3β in this paradigm of alcohol exposure, it is necessary to determine whether blocking GSK3β activation is sufficient to protect the hippocampus from alcohol-induced damage.

In summary, this paradigm of binge alcohol exposure reduced neurogenesis and increased neurodegeneration in the hippocampus of rats which was accompanied with the activation of microglia. More importantly, the binge alcohol exposure impaired spatial learning and memory, and activated GSK3β by inducing dephosphorylation at Ser9. Since GSK3β is a known mediator of alcohol neurotoxicity in the developing brain, it may play a role in alcohol-induced damage in the adolescent hippocampus. Future studies are necessary to determine whether GSK3β inhibitors are able to protect alcohol-induced damage to the adolescent hippocampus and related behavioral deficits.

## Materials and Methods

### Alcohol exposure paradigm

35-day-old Sprague-Dawley rats were obtained from Shanghai SLAC Laboratory Animal Co. Ltd (Shanghai, China). The experiments were approved by the Institutional Animal Care and Use Committee of the Shanghai University of Traditional Chinese Medicine. All methods for rats studies involving rat samples were performed in accordance with the relevant guidelines and regulations. A well-established paradigm of binge alcohol exposure was used in this study^33–35^. Briefly, rats were maintained on 12 hrs light/dark cycle with *ad libitum* access to food and water except during the alcohol exposure. Food was removed during the period of alcohol treatment, although water was freely available. There were two group of animals and each groups had 35 rats. Rats were gavaged with an alcohol diet (25% alcohol w/v in nutritionally complete diet (Abbott Laboratories, Columbus, OH) (ETOH) or isocaloric control diet (CT) every 8 hrs for 4 days. After a priming dose of 5 g/kg, subsequent doses were determined using a six-point behavioral intoxication scale: 0, normal; 1, hypoactive; 2, ataxia; 3, ataxia with dragging abdomen and/or delayed righting reflex; 4, loss of righting reflex; 5, loss of eye blink reflex. Each score was associated with a dose of alcohol between 0 and 5 g/kg.

### Immunohistochemistry and immunofluorescence staining

For immunohistochemical analysis of brain tissues, Rats were euthanized by intracardiac perfusion with ice-cold phosphate buffered saline (PBS), followed by 4% paraformaldehyde in 0.1 M phosphate buffer, pH 7.4, under anesthesia with sodium pentobarbital. Brain was removed immediately after the perfusion and post-fixed for 12 hours in 4% paraformaldehyde at 4 °C. Brain was sectioned at a thickness of 25 µm. The sections were rinsed in PBS, incubated with 0.3% hydrogen peroxide, blocked by the incubation with 1% bovine serum albumin at 37 °C for 1 hour, and then incubated overnight at 4 °C with a primary antibody. Mouse anti-neuron-specific nuclear protein (NeuN) antibody and biotin-labeled secondary antibodies were obtained from Chemicon International Inc. (Temecula, CA, USA). Mouse anti-glial fibrillary acidic protein (GFAP) antibody was obtained from Sigma Chemical Co. Goat anti-Doublecortin (C-18) antibody was obtained from Santa Cruz Biotechnology, Inc. (Santa Cruz, CA, USA). Anti-IBA1 antibody was purchased from Wako (Osaka, Japan). Rabbit anti-phospho-GSK-3β (Ser9) (5B3) was obtained from Cell Signaling Technology (Beverly, MA); Rabbit anti-phospho-GSK3β (Tyr216) antibody was obtained from Abcam Co. (Cambridge, MA, USA). After rinsing in PBS, the sections were then incubated with appropriate biotinylated secondary antibodies at 37 °C for 1 hour. Sections were then incubated with avidin-biotin-peroxidase complex for 1 hour, rinsed in PBS and developed in 0.05% diaminobenzidine (DAB) with 0.003% H_2_O_2_ in PBS. DAB was obtained from Vector Laboratories Inc. (Burlingame, CA, USA). All antibodies were diluted in 1% bovine serum albumin in PBS. Negative controls were performed by the incubation of pre-immune IgG. The bright field images were taken on a BX51 Olympus microscope (Olympus Corporation, Tokyo, Japan).

For immunocytofluorescence staining, sections were rinsed in PBS, blocked by incubation with 1% bovine serum albumin at 37 °C for 1 hour, and then incubated overnight at 4 °C with primary antibodies. The sections were incubated with appropriate PE-secondary antibodies at 37 °C for 1 hour. Immunofluorescent images were recorded using a Zeiss LSM 510 Meta confocal microscope (Carl Zeiss MicroImaging Inc., Thornwood, NY, USA).

For the quantification, five sections from each rat (5 rats for each group) were used for cell counting. Cells were counted by using ImageJ (US National Institutes of Health) in a designated area. Data represent mean ± SD.

### Sample preparation and immunoblotting

After treatment, rats were anesthetized by intraperitoneal injection of sodium pentobarbital (2 mg/10 g body weight), and the hippocampus was immediately dissected. The tissues were frozen in liquid nitrogen and stored at −80 °C. Proteins were extracted as previously described^47^. Briefly, tissues were homogenized in an ice-cold lysis buffer containing 50 mM Tris-HCl (pH 7.5), 150 mM NaCl, 1 mM EGTA, 1 mM PMSF, 0.5% NP-40, 0.25% SDS, 5 μg/ml leupeptin, and 5 μg/ml aprotinin. Homogenates were centrifuged at 20,000 g for 30 min at 4 °C, and the supernatant fraction was collected.

The immunoblotting procedure has been previously described^48^. Briefly, aliquots of the protein samples (30 μg) were separated on a SDS-polyacrylamide gel by electrophoresis. The separated proteins were transferred to nitrocellulose membranes. The membranes were blocked with either 5% BSA or 5% nonfat milk in 0.01 M PBS (pH 7.4) and 0.05% Tween-20 (TPBS) at room temperature for 1 hour. Subsequently, the membranes were probed with primary antibodies directed against target proteins overnight at 4 °C. Rabbit anti-phospho-GSK-3β (Ser9) (5B3) was obtained from Cell Signaling Technology (Beverly, MA); Rabbit anti-phospho-GSK3β (Tyr216) antibody was obtained from Abcam Co. (Cambridge, MA, USA). Anti-glyceraldehyde-3-phosphate dehydrogenase (GAPDH) antibody was obtained from Kangcheng Bio-tech Inc. (Shanghai, China). After three quick washes in TPBS, the membranes were incubated with a secondary antibody conjugated to horseradish peroxidase (Amersham, Arlington Heights, IL). The immune complexes were detected by the enhanced chemiluminescence method (Amersham, Arlington Heights, IL). The density of immunoblotting was quantified with the software Quantity One (Bio-Rad, Hercules, CA).

### Electron microscopy

The electron microscopy protocol as described before^49^, briefly, rats were transcardially perfused with 4% paraformaldehyde in 0.1 M phosphate buffer, pH 7.4, and maintained at 4 °C. Brain tissue was removed immediately after the perfusion and post-fixed for 12 hours in 4% paraformaldehyde. The tissue was sectioned at a thickness of 100 µm horizontally with a vibratome, and then postfixed overnight in 2.5% glutaraldehyde, washed in 0.1 M phosphate buffer, postfixed in osmium tetroxide for 1 hour, dehydrated in ascending concentrations of alcohol and then acetone, and embedded in Epoxy resin. Serial sections of 40 nm thickness were collected on single-slot grids, and then contrasted by incubating for 35 min in 5% uranyl acetate solution, followed by 25 min in a Reynolds solution. Serial images of the labeled structures were then collected with a SIS MegaView III high resolution CCD camera mounted on a JEOL 100 CXII transmission electron microscope (JEOL USA, Inc. Peabody, MA) at a 19,000× magnification with a filament voltage of 80 kV.

### Morris water maze

The Morris water maze (MWM) was used to assess the learning and memory ability of the rat. The MWM was performed as previously described^50,51^. Briefly, the water maze, a black circular pool of 120 cm in diameter and 50 cm in height, was filled with water to a depth of 32 cm at 25 °C. A circular transparent plexiglas platform, 8 cm in diameter, was permanently placed in the middle of the northeast quadrant, 40 cm into the pool, and 0.5 cm below the water surface. The experiment started on the third day after the last gavage, and the trial sessions for place navigation within the MWM were continued for 5 sessions. Each session included one search for the platform from different starting positions of the four quadrants. The sequential order of the searches for the platform was randomly selected from day to day, but all rats at each day had the same order. The performance of the rats was monitored with an overhead video camera connected to an image analyzer (Jiangliang Bioinstrumentation Ltd, Shanghai, China). Experiments were performed between 8 a.m. and 2 p.m.

The time to reach the platform was recorded in each trial, with a maximal time limit of 60 seconds. If the platform was not found within the set time, the computer stopped tracking and recorded the time as 60 seconds. If the rat found the platform within 60 seconds, it was allowed to stay on it for 10 seconds; otherwise, the rat was gently guided to find the platform by the experimenter and allowed to remain on the platform for 10 seconds. The time to reach the platform (latency to find the platform) and the distance of swimming were measured with a computerized tracking system. At the end of the training period, the rats were tested on a spatial probe trial in which the platform was removed. The rats were allowed to swim freely for 60 seconds. The percentage of time spent in the target quadrant and the number for these rats to cross the area in which the original platform was placed were recorded.

### Open field test

In order to assess any possible effects of alcohol treatments on the spontaneous locomotor activity, the open field test was performed. The protocol as described before^52,53^. Briefly, twenty-four hours after the last alcohol treatment, 20 rats (n = 10, each group) were individually placed into an open field (automated TruScan test arena, Coulbourn Inc., PA) for 10 min in normal illumination. The test arenas are equipped with sensor rings that sense in the X-Y dimension (for crossings) and in the Z plane vertical dimension (for rearing). Each arena is linked via a station interface box to a computer. Overall activity in the box was measured as well as the amount of time and distance traveled in the center area of the maze. This paradigm is based on the idea that rodents will naturally prefer to be near a protective wall rather than exposed to danger out in the open.

### Statistical analysis

Statistical analysis was assessed by ANOVA followed by Student–Newman–Keuls analyses. An unpaired t test was used for the analysis of quantitative data of cell counting. Data were presented as means ± S.E.M. Difference in which p < 0.05 was considered statistically significant.

## Electronic supplementary material

Supplementary Information
