# Supplementary material for: Binge Alcohol Exposure Causes Neurobehavioral Deficits and GSK3β Activation in the Hippocampus of Adolescent Rats
Source: Sci Rep. 2018 Feb 15;8:3088. doi: 10.1038/s41598-018-21341-w (PMC5814471; doi:10.1038/s41598-018-21341-w)

Binge Alcohol Exposure Causes Neurobehavioral Deficits and GSK3β Activation in the Hippocampus of Adolescent Rats

Zhe Ji[1,2], Lin Yuan[1], Xiong Lu[1], Hanqing Ding[1], Jia Luo[1,3], Zun-Ji Ke[1]

Figure 6:

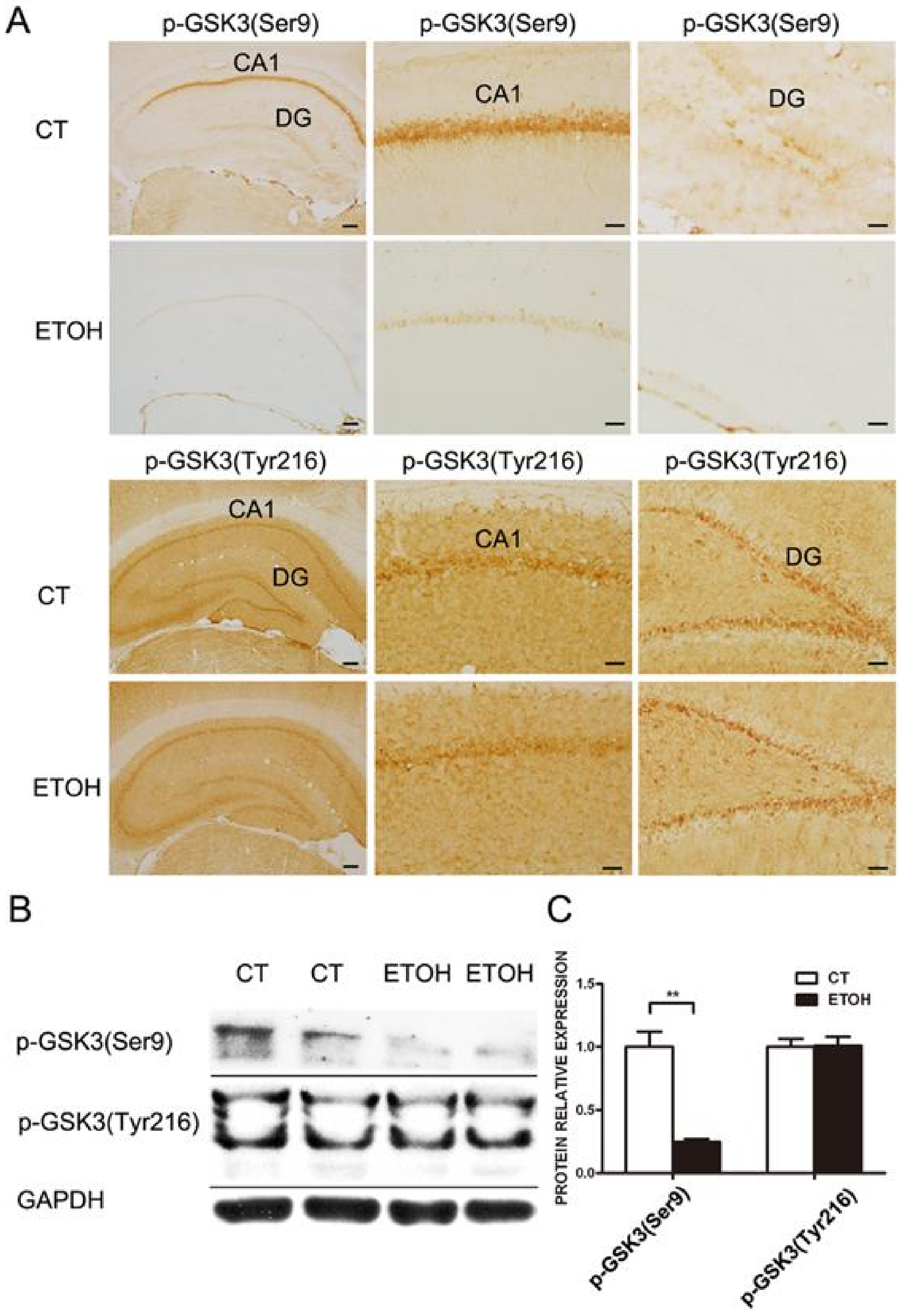

Figure 6A

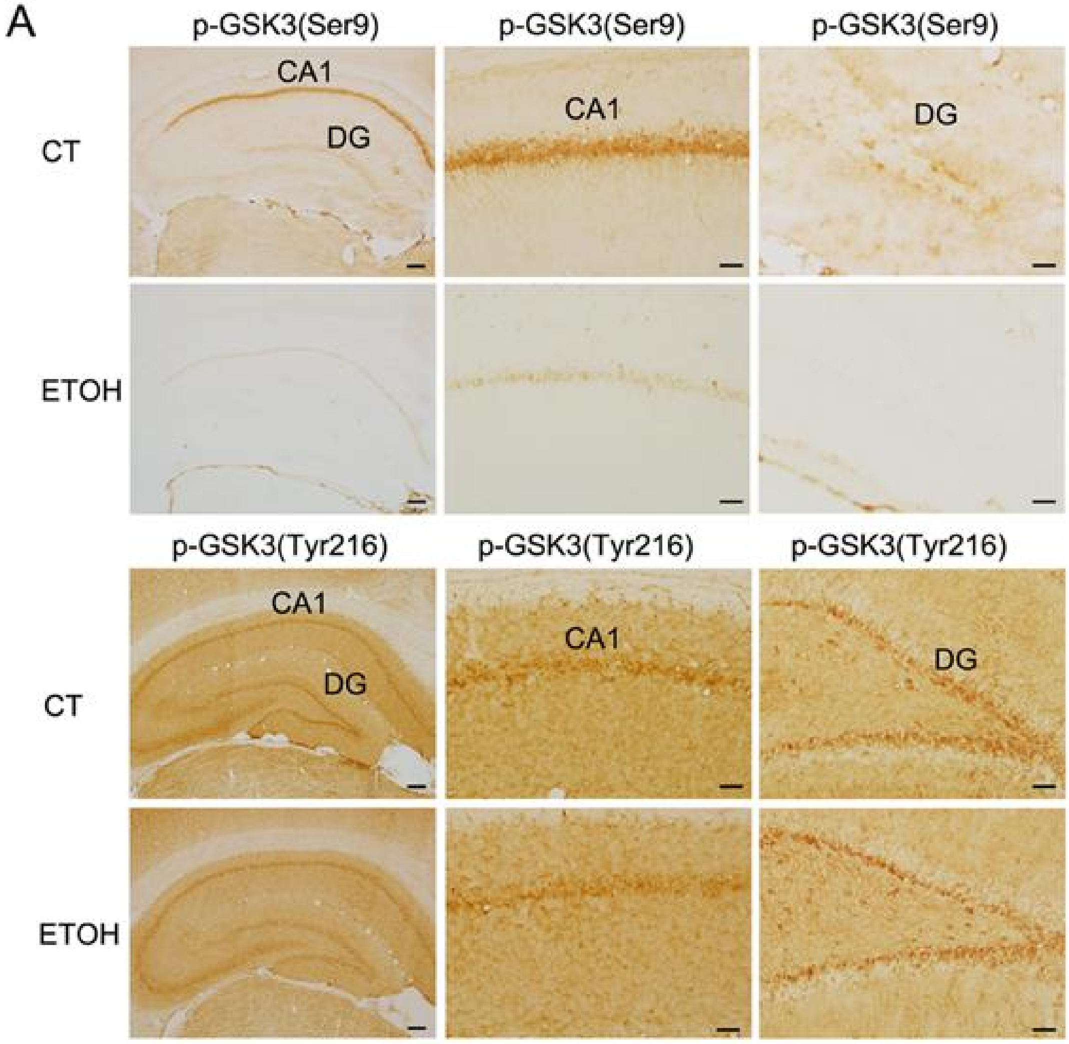
A
p-GSK3(Ser9)
p-GSK3(Ser9)
p-GSK3(Ser9)
CA1
DG
CA1
DG
CT
ETOH
p-GSK3(Tyr216)
p-GSK3(Tyr216)
p-GSK3(Tyr216)
CA1
DG
CA1
DG
CT
ETOH

Figure 6B

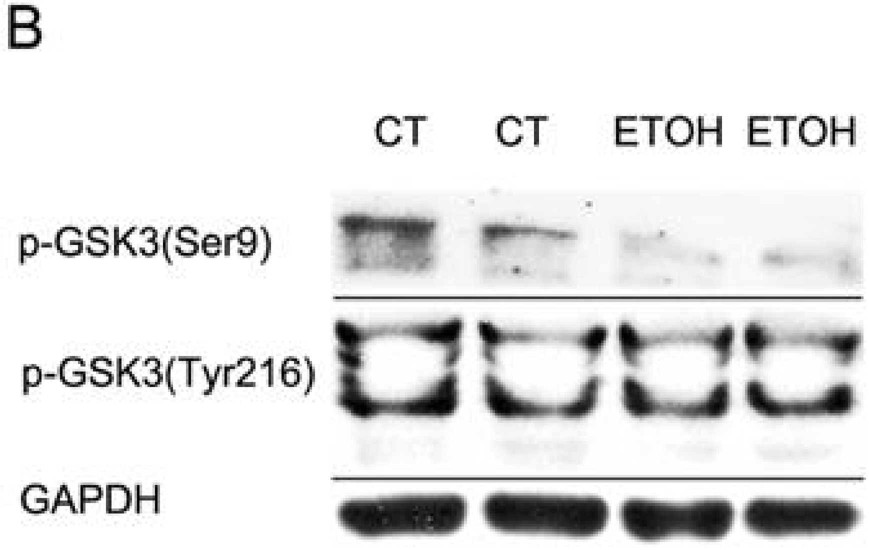

p-GSK3(Ser9)

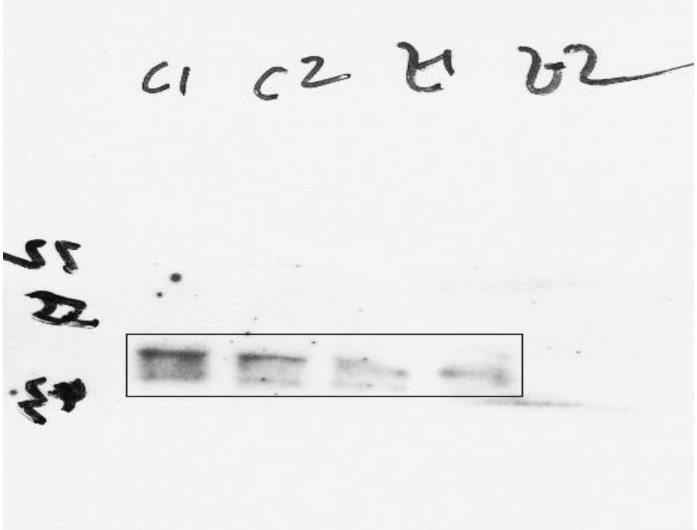

p-GSK3(Thr216)

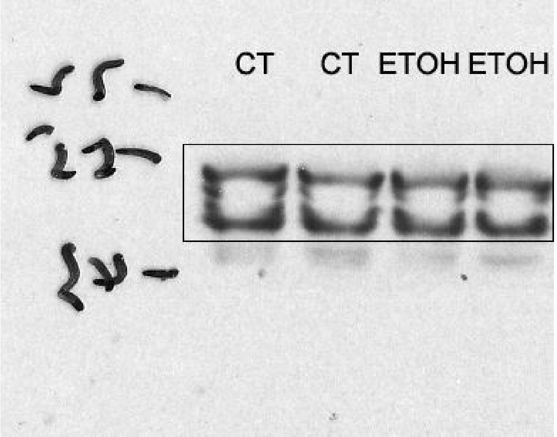

GAPGH

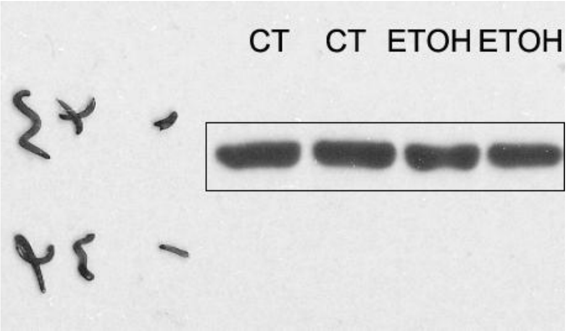

Figure 6C

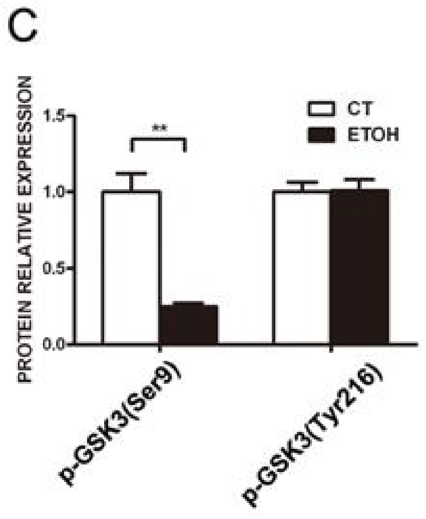

Supplement: Supplementary file 1 — Supplementary Information [file 41598_2018_21341_MOESM1_ESM.pdf]
